# Tracing the haze: satellite-based assessment of stubble burning and air quality in Delhi

**DOI:** 10.1007/s11869-026-01885-x

**Published:** 2026-01-21

**Authors:** Tharani Kotrike, Venkataramana Sridhar

**Affiliations:** 1 Department of Civil Engineering, G Pulla Reddy Engineering College, Kurnool, India; 2 Department of Biological Systems Engineering, Virginia Polytechnic Institute and State University, Blacksburg, VA 24061 USA

**Keywords:** AOD, Active fire events, NOAA-HYSPLIT trajectory, NDVI, Stubble burning

## Abstract

New Delhi, the capital city of India, routinely records hazardous fine-particle concentrations during the post-monsoon season, yet the quantitative link between regional crop-residue burning and episodic haze remains contested. This study integrates multi-sensor satellite products with atmospheric trajectory modelling to attribute the late-October–early-November aerosol enhancement over the capital during 2020–2024. Columnar aerosol optical depth (AOD) at 550 nm was extracted from MODIS Terra–Aqua (10 km); active-fire detections were taken from VIIRS S-NPP (375 m); harvest dynamics were approximated from MODIS NDVI (250 m); and 120 h forward air-mass trajectories at 500–1000 m a.g.l. were generated with NOAA-HYSPLIT driven by GDAS 1° fields. Seasonal-trend decomposition and Theil–Sen statistics revealed a consistent AOD surge of 0.35 ± 0.06 above pre-monsoon levels (*p* < 0.05). Punjab contributed 90% of regional fire counts in 2020 but only 40% in 2024, whereas Haryana showed a marginal decline. Daily fire counts within Punjab–Haryana explained 78% of Delhi AOD variance during October–November (*r* = 0.78, p ≪ 0.01). NDVI differencing confirmed harvest-related vegetation loss across > 90% of cropland pixels in week 43 each year. Cluster analysis indicated that 60% of trajectories originating overactive-burn zones intersected Delhi within 36 h, increasing the probability of AOD > 1.2 by a factor of five. These convergent lines of evidence identify stubble combustion as the primary driver of Delhi’s recurring autumn haze. Accelerated deployment of in-situ straw incorporation, baler-mulcher systems, and regional burning-ban enforcement, supported by real-time satellite surveillance, is recommended to achieve National Clean Air Programme particulate-matter targets and to safeguard regional public health. Economic co-benefits are anticipated through fuel savings, improved soil organic carbon, and rural air-quality gains across the Indo-Gangetic Plain.

## Introduction

Rapid urban expansion, population growth, and intensified industrial activity have pushed ambient air quality in many Indian megacities beyond safe limits. Delhi National Capital Region (NCR) registers some of the highest fine-particulate (PM₂.₅ and PM₁₀) levels worldwide, creating chronic public-health risks and economic losses (Nair et al. 2021; Pandey et al. 2021). The burden is driven by a complex mixture of local emissions—traffic, residential biomass, construction dust—and regional inflow of gaseous pollutants (SO₂, NO₂, CO, volatile organic compounds) and aerosols (Choudhary et al. 2023; Kumar et al. 2020). These pollutants alter the surface radiative balance, reduce visibility, and have been linked to excess mortality (Feng And Christopher 2013).

Atmospheric aerosols consist of natural and anthropogenic particles, including soil dust, sea salt, sulphates, black carbon, organic compounds, and secondary inorganics (Hussain et al. 2023; Mahato et al. 2023). In the Indo-Gangetic Plain, one of the most important anthropogenic contributors is large-scale crop-residue burning (CRB) (Vijaykumar et al. 2024). Farmers in Punjab and Haryana routinely ignite paddy straw during October–November and wheat straw during April–May to clear fields between cropping cycles (Roy et al. 2022; Sarkar et al. 2018). In 2023 alone, an estimated 500 Mt of residue were burned, producing extensive smoke plumes advected towards Delhi by prevailing north-westerlies (Lan et al. 2022). Under post-monsoon temperature inversions and a shallow planetary boundary layer, these plumes can transform Delhi’s atmosphere into a “toxic gas chamber,” sustaining “poor” or “very poor” air-quality categories for days (Goel et al. 2021; Kulkarni et al. 2020).

Ground-based monitors offer high-frequency observations but provide limited spatial coverage and cannot resolve the origin of transported pollutants. Satellite remote sensing has therefore become indispensable for regional attribution studies (Kumar et al. 2020; Kotrike et al. 2021; Jodhani et al. 2024a). Instruments such as MODIS, VIIRS, MISR, and AVHRR deliver daily aerosol optical depth (AOD) retrievals, fire detections, and vegetation indices with kilometer-scale resolution. MODIS 550 nm AOD, in particular, has been widely adopted as a proxy for column-integrated particulate loading in urban environments (Kumar et al. 2024). When combined with Lagrangian dispersion tools such as the National Oceanic and Atmospheric Administration Hybrid Single-Particle Lagrangian Integrated Trajectory model (NOAA-HYSPLIT), satellite data enable quantitative source–receptor analysis of CRB episodes.

Despite multiple case studies, the strength of the CRB signal relative to other sources remains a matter of debate, complicated by year-to-year variability in residue-management policies, meteorology, and emissions inventories (Mogno et al. 2023). To craft evidence-based interventions under the National Clean Air Programme (NCAP), a multiyear, high-resolution examination of Delhi’s post-monsoon aerosol climatology is required (Mangaraj et al. 2025; Abdurrahman et al. 2020). Similar multi-sensor remote sensing frameworks have been effectively applied to study extreme weather and hydrometeorological events across Indian cities, reinforcing the utility of satellite-derived indicators for climate and air quality analyses (Kotrike et al. 2024). Integrating remote sensing with ground observations improves spatial attribution of environmental variables, as established in comparative precipitation and soil moisture studies over in several regions including India (Sridhar et al. 2013; Setti et al. 2020a).

The present work aims to (1) map the spatiotemporal variability of aerosols over Delhi between 2020 and 2024, (2) perform a statistical assessment of MODIS 550 nm AOD trends, (3) identify extreme active-fire periods in Punjab–Haryana and relate them to Delhi aerosol peaks, and (4) verify the transport pathway of smoke using NOAA-HYSPLIT forward trajectories. By integrating multi-sensor satellite products with trajectory analysis, the study provides an updated, quantitative attribution of autumn haze episodes and offers actionable insights for regional air-quality management.

## Study area

**Fig. 1:**
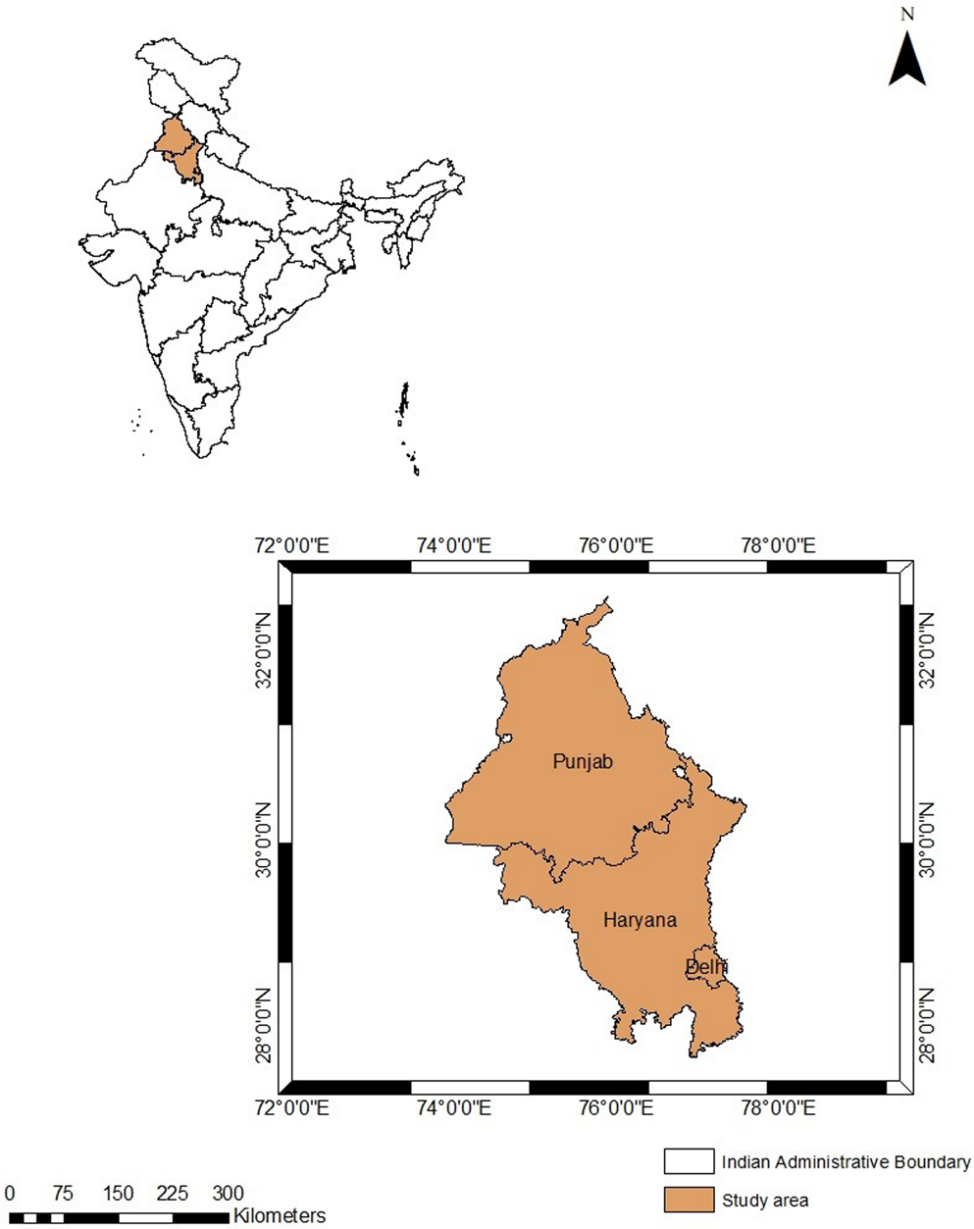
Study Area Map Showing Delhi and Adjacent Regions of Punjab and Haryana Affected by Stubble Burning

The analysis covers three administrative units in northern India—Punjab, Haryana, and the National Capital Territory (NCT) of Delhi—whose geographic setting is illustrated in Fig. 1. Together they form the principal upwind corridor that influences Delhi’s post-monsoon air quality.

Punjab (50, 362 km²) occupies the north-western Indo-Gangetic Plain. It is flanked by the Union Territory of Jammu & Kashmir to the north, Himachal Pradesh to the north-east, Haryana to the south-east, and Rajasthan to the south-west. The state consists almost entirely of alluvial plains dissected by five perennial rivers and an extensive canal-irrigation network. Mean elevation is ~ 300 m a.s.l., varying from > 500 m along the Shivalik foothills in the north-east to ~ 180 m in the arid south-west, where the landscape grades into the Thar Desert. Punjab experiences a subtropical climate with large seasonal temperature amplitudes: mean minima of ≈ 12 °C in January and mean maxima approaching 47 °C in June. The predominance of rice–wheat double cropping and strict turnaround times encourage large-scale crop-residue burning each October–November.

Haryana (4.42 million ha) is land-locked between 27°39′–30°35′ N and 74°28′–77°36′ E, bordering Rajasthan to the west–south, Punjab and Himachal Pradesh to the north, and Uttar Pradesh to the east. It surrounds Delhi on three sides (north, west, south). Most of the state lies on the fertile Ghaggar plain—part of the Indo-Gangetic fluvial system—supporting intensive mechanized agriculture that likewise relies on open-field burning to clear paddy straw.

Delhi–NCT (1 483 km²; 28.61° N, 77.23° E) comprises the national capital, New Delhi, and surrounding urban districts. Topography is defined by the Yamuna River floodplain and the Delhi Ridge, an extension of the Aravalli hills. The city has a hot semi-arid climate: mean daily highs exceed 39 °C during March–June, while November–January lows average ~ 7 °C. Delhi routinely records hazardous particulate concentrations; Air Quality Index values remain *Moderate* (101–200) through most of the year but deteriorate to *Very Poor* (301–400), *Severe* (401–500), or *Hazardous* (> 500) during October–December (Horn and Dasgupta 2024). The seasonal deterioration coincides with north-westerly transport of smoke from crop-residue fires in Punjab and Haryana under stagnant boundary-layer conditions, making the tri-state region a natural focus for source–receptor analysis of autumn haze.

## Datasets and pre-processing

This study utilizes multiple remote sensing datasets to assess aerosol variability, active fire locations, and vegetation phenology across Punjab, Haryana, and Delhi during 2020–2024. The data include MODIS Aerosol Optical Depth (AOD), VIIRS active fire detections, MODIS NDVI composites, and NOAA HYSPLIT forward trajectories. A summary of the datasets is presented in Table 1.

**Table 1:** Summary of datasets used in the study, including satellite products, Temporal coverage, Spatial resolution, and data sources. The datasets were integrated to assess the impact of crop residue burning on air quality in Delhi using aerosol, vegetation, fire, and atmospheric transport indicators

Dataset	Description	Temporal Resolution	Spatial Resolution	Source
MODIS AOD (MOD04_L2)	Daily aerosol optical depth at 550 nm from Terra and Aqua satellites	Daily	10 km × 10 km	NASA LAADS DAAC
VIIRS S-NPP	Thermal anomalies indicating active fire locations	Daily	375 m	NASA Firms
MODIS NDVI (MOD13A1)	16-day composite of Normalized Difference Vegetation Index	16-day composite	500 m	NASA Earthdata
NOAA HYSPLIT	Forward and backward air-mass trajectories using GDAS meteorological fields	6-hourly initialization	1° × 1°	NOAA Ready

### Aerosol optical depth

Satellite-based AOD products serve as a critical resource for understanding the spatiotemporal distribution of aerosols. In this study, MODIS Level 2 Collection 6.1 AOD data (MOD04_L2) at 550 nm were used. These data are retrieved from the Moderate Resolution Imaging Spectroradiometer (MODIS) aboard the Terra and Aqua satellites, which operate at an altitude of approximately 705 km and have local overpass times of 10:30 IST (Terra) and 13:30 IST (Aqua), respectively. MODIS provides AOD measurements at a spatial resolution of 10 km × 10 km with near-global coverage.

The retrieval algorithm estimates top-of-atmosphere radiances at 470 nm and 670 nm using radiative transfer look-up tables, and the Angström exponential law is then applied to derive AOD at 550 nm (Vijaykumar et al., 2018; Kotrike et al. 2021). These data allow for daily monitoring of aerosol loadings, making them valuable for air quality analysis over Delhi and its surroundings. According to Tong et al. 2020 the AOD obtained from MODIS is in good agreement with AERONET AOD in Asia.

###  Active fire data

To identify fire activity in the region, active fire data from the Visible Infrared Imaging Radiometer Suite (VIIRS) onboard the Suomi-NPP satellite were used. VIIRS provides data across 22 spectral bands, ranging from visible to longwave infrared, with two spatial resolutions: 375 m (I-band) and 750 m (M-band).

For this study, thermal anomaly detections at 375 m resolution were utilized to estimate active fire locations. These fire detections are based on thermal infrared brightness temperatures and contextual tests for distinguishing fire pixels from background surfaces (Schroeder et al. 2014). VIIRS fire data offer high spatial and temporal resolution, allowing accurate identification of stubble-burning hotspots across Punjab and Haryana during the peak burning seasons of October–November (paddy residue) and April–May (wheat residue).

### MODIS NDVI

Vegetation condition and phenological changes were assessed using the MODIS 16-Day NDVI composite product (MOD13A1), which provides a spatial resolution of 500 m. NDVI serves as a robust indicator of vegetation health, derived from red and near-infrared reflectance that respond to leaf chlorophyll content, canopy structure, and leaf area. NDVI data have been proven effective proxies for tracking crop conditions and inferring biomass changes linked to harvest cycles (Anand et al. 2024).

The MOD13A1 product uses daily, atmosphere-corrected, bidirectional surface reflectance and applies a compositing algorithm to retain only high-quality pixels. Low-quality observations affected by clouds or poor view angles are discarded based on MODIS-specific quality assurance (QA) measures. From the remaining high-quality data, the pixel with the best viewing geometry is selected to represent each 16-day period.

In this study, NDVI was used to identify post-monsoon vegetation decline in the last week of October, corresponding to paddy harvest and subsequent residue burning. A reduction in NDVI across > 90% of cropland pixels served as a proxy for biomass removal prior to fire activity.

## Approach

**Fig. 2:**
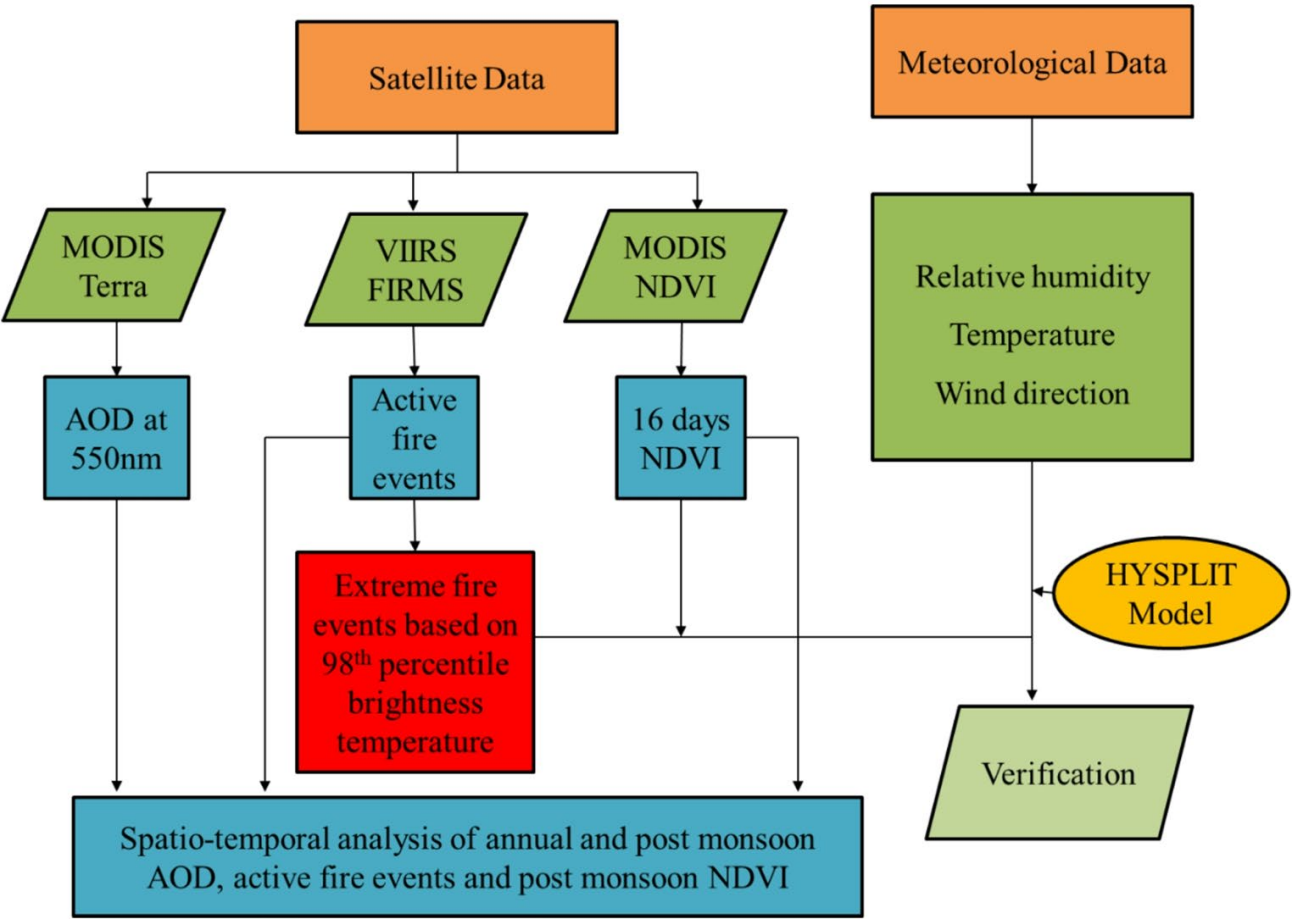
Methodological framework adopted for the study, integrating MODIS AOD, VIIRS active fire data, MODIS NDVI, and NOAA HYSPLIT trajectory analysis to evaluate the spatiotemporal impact of stubble burning on air quality in Delhi from 2020 to 2024

The workflow adopted to quantify the influence of crop-residue burning on Delhi’s aerosol loading is summarised in Fig. 2 and consists of three sequential components: (i) Spatio-temporal analysis of aerosol optical depth (AOD) and vegetation state, (ii) statistical identification of extreme fire days, and (iii) Lagrangian forward-trajectory diagnosis of smoke transport.

### Satellite-data processing and exploratory analysis

**AOD mosaics** – Level-2 MODIS Terra-Aqua 550 nm AOD scenes (Collection 6.1) for January 2020–December 2024 were downloaded from the LAADS-DAAC portal. Pixels flagged as cloud-contaminated or “no-data” were discarded. The remaining retrievals from the two overpasses were averaged to obtain a single daily field at 10 km × 10 km. Annual means were calculated on a pixel-by-pixel basis, producing five gridded maps that reveal spatial gradients across Punjab, Haryana, and Delhi as well as inter-annual changes. A second aggregation targeted only the post-monsoon season (weeks 40–48) to isolate the period most affected by stubble burning.

**Vegetation phenology** – MODIS 16-day NDVI composites (MOD13A1, 500 m) were re-projected to a common WGS-84 grid. Quality-assurance (QA) flags were applied to mask cloud, cirrus, and high-view-angle pixels. The time series was differenced between week 41 and week 44 each year to map abrupt declines in canopy greenness that coincide with paddy harvest and field clearing.

**Active-fire catalogue** – VIIRS I-band 375 m thermal-anomaly detections were ingested from NASA FIRMS. Only “Nominal” and “High” confidence records within Punjab, Haryana, and Delhi were retained. For each detection, the brightness-temperature (BT) attribute was used as a proxy for combustion intensity. While recognizing BT does not directly represent combustion intensity, it was chosen as the primary indicator because this study focuses on active fire detection.

### Identification of extreme fire events

Daily VIIRS detections were spatially joined to district polygons in ArcMap 10.2 and summed to produce an area-normalized fire count. Brightness-Temperature (BT) values for all detections in a given calendar year were sorted in ascending order in Microsoft Excel; the 98th-percentile BT was taken as an empirical threshold. Any detection with BT > T_98_ was labelled an “extreme fire.” A day was classified as an extreme-fire day if more than 10 such detections occurred within Punjab-Haryana on that date. The procedure yielded a concise list of high-intensity episodes (Table 2) that anchor the trajectory analysis.

**Table 2:** Summary of extreme fire events in the study area in 2024, identified based on the 98th percentile threshold of VIIRS active fire count data. The table highlights the dates and intensity of peak fire activity, primarily concentrated in Punjab and Haryana during the post-monsoon season

S.No	Date	Brightness Temperature(K)	S.No	Date	Brightness Temperature(K)
1	14-Oct-2024	341.72	24	06-Nov-2024	347.28
2	17-Oct − 2024	344.74	25	08-Nov-2024	348.71
3	19- Oct-2024	349.71	26	08-Nov-2024	343.39
4	27- Oct-2024	349.84	27	08-Nov-2024	342.69
5	29- Oct-2024	343.42	28	08-Nov-2024	351.83
6	29- Oct-2024	343.06	29	08-Nov-2024	342.73
7	29- Oct-2024	346.52	30	13-Nov-2024	347.64
8	29- Oct-2024	346.69	31	15-Nov-2024	345.64
9	29- Oct-2024	344.49	32	18-Nov-2024	349.52
10	30- Oct-2024	360.17	33	18-Nov-2024	352.99
11	31- Oct-2024	342.21	34	18-Nov-2024	348.93
12	31- Oct-2024	352.65	35	18-Nov-2024	341.95
13	31- Oct-2024	353.65	36	18-Nov-2024	386.74
14	31-Oct-2024	353.08	37	18-Nov-2024	344.49
15	01-Nov-2024	343.06	38	18-Nov-2024	353.39
16	03-Nov-2024	345.05	39	18-Nov-2024	344.42
17	03-Nov-2024	342.55	40	21-Nov-2024	345.81
18	03-Nov-2024	346.62	41	21-Nov-2024	342.51
19	03-Nov-2024	341.95	42	23-Nov-2024	349.80
20	03-Nov-2024	343.24	43	23-Nov-2024	342.32
21	03-Nov-2024	346.55	44	25-Nov-2024	345.54
22	05-Nov-2024	341.91	45	14-Dec-2024	355.14
23	05-Nov-2024	342.21	46	14-Dec-2024	363.92

### Forward-trajectory diagnosis (NOAA-HYSPLIT)

For every extreme-fire day, 120-h forward air-mass trajectories were generated at 6-h initializations (00, 06, 12, 18 UTC) with the Hybrid Single-Particle Lagrangian Integrated Trajectory (HYSPLIT) model, using 1° × 1° GDAS meteorological fields. Start heights were fixed at 500 m and 1000 m a.g.l., representative of Delhi’s mixed-layer top during the post-monsoon season. Trajectories were overlaid on the VIIRS fire mask to determine the fraction intersecting the Punjab-Haryana burn corridor.

Model output was verified against concurrent meteorological observations (surface temperature, relative humidity, and prevailing wind direction) from the India Meteorological Department’s stations across NCR. In addition, 10-day AOD composites following each extreme-fire day were examined to detect monotonic aerosol build-up over Delhi, while near-surface temperature anomalies were used as an indirect indicator of elevated absorbing-aerosol (black-carbon) load.

By integrating high-resolution satellite observations with trajectory physics, this approach links emission intensity, transport pathways, and downwind aerosol enhancements, thereby providing a robust attribution of Delhi’s recurrent post-monsoon haze to crop-residue burning in Punjab and Haryana.

## Results and discussions

### Annual spatio-temporal distribution of AOD

**Fig. 3:**
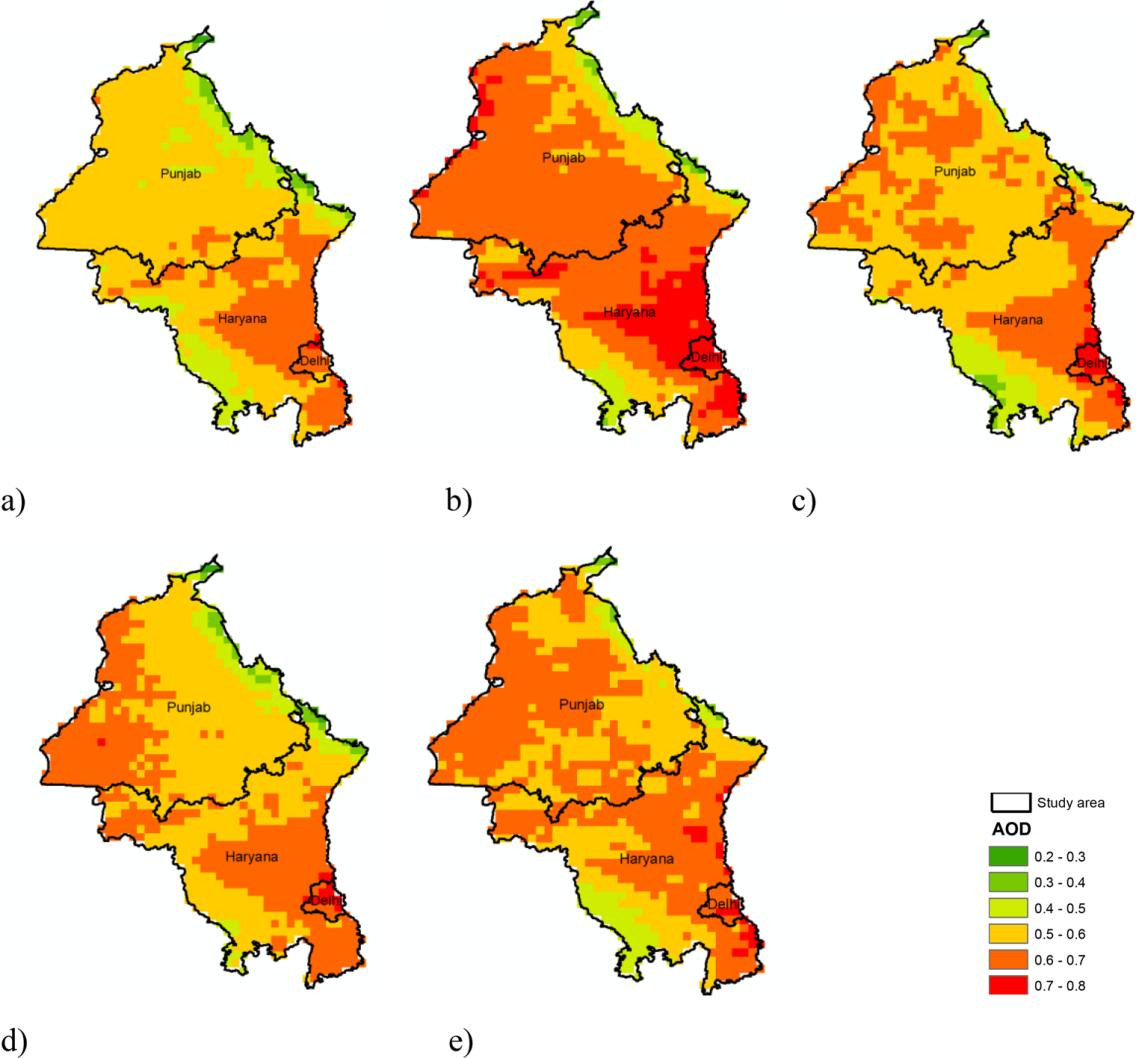
Spatiotemporal distribution of annual mean Aerosol Optical Depth (AOD) over Delhi and surrounding regions for the years (**a**) 2020, (**b**) 2021, (**c**) 2022, (**d**) 2023, and (**e**) 2024. The maps illustrate interannual variability in aerosol concentrations, highlighting persistent high AOD levels and changes in spatial patterns associated with crop residue burning

Land use and climate dynamics have been shown to shape aerosol behavior and surface energy balances in other Indian basins, which may indirectly influence transport and retention of particulate matter (Loukika et al. 2025). Mean 550 nm AOD for 2020–2024 (Fig. 3a–e) ranges from 0.2 to 0.7 across Punjab, Haryana, and Delhi. In 2020 roughly 5.7 × 10^5^ ha—principally central Punjab and adjoining Haryana—registered values between 0.50 and 0.60, while Delhi recorded the highest class (0.70–0.80) over ~ 2 × 10^3^ ha. The regional mean climbed in 2021, with 0.60–0.70 AOD expanding to ~ 5.6 × 10^5^ ha and the 0.70–0.80 class engulfing all of Delhi plus 1.21 × 10^5^ ha of southern-eastern Haryana.

AOD intensity moderated in 2022: the 0.50–0.60 class shrank to ~ 4.9 × 10^5^ ha, although Delhi retained persistent 0.70–0.80 values (0.19 × 10^5^ ha). In 2023 the 0.60–0.70 patch over Punjab migrated westward and total 0.50–0.60 coverage fell to ~ 4.6 × 10^5^ ha, yet Delhi again exhibited the highest class, albeit over a reduced 0.089 × 10^5^ ha. AOD rebounded in 2024 across Punjab–Haryana but declined over Delhi, a contrast tentatively attributed to (i) the Minimum Support Price (MSP)–linked offtake of rice straw reducing local burning near Delhi and (ii) the findings of the survey reported by Tewari et al. (2025) wherein the farmers are aware of the health and environmental risks of stubble burning, adoption of sustainable practices is mainly influenced by their health concerns, perceived risks, and social networks rather than their technical coping capacity or supply chain access.

### Post-monsoon (Oct–Nov) distribution of AOD

**Fig. 4:**
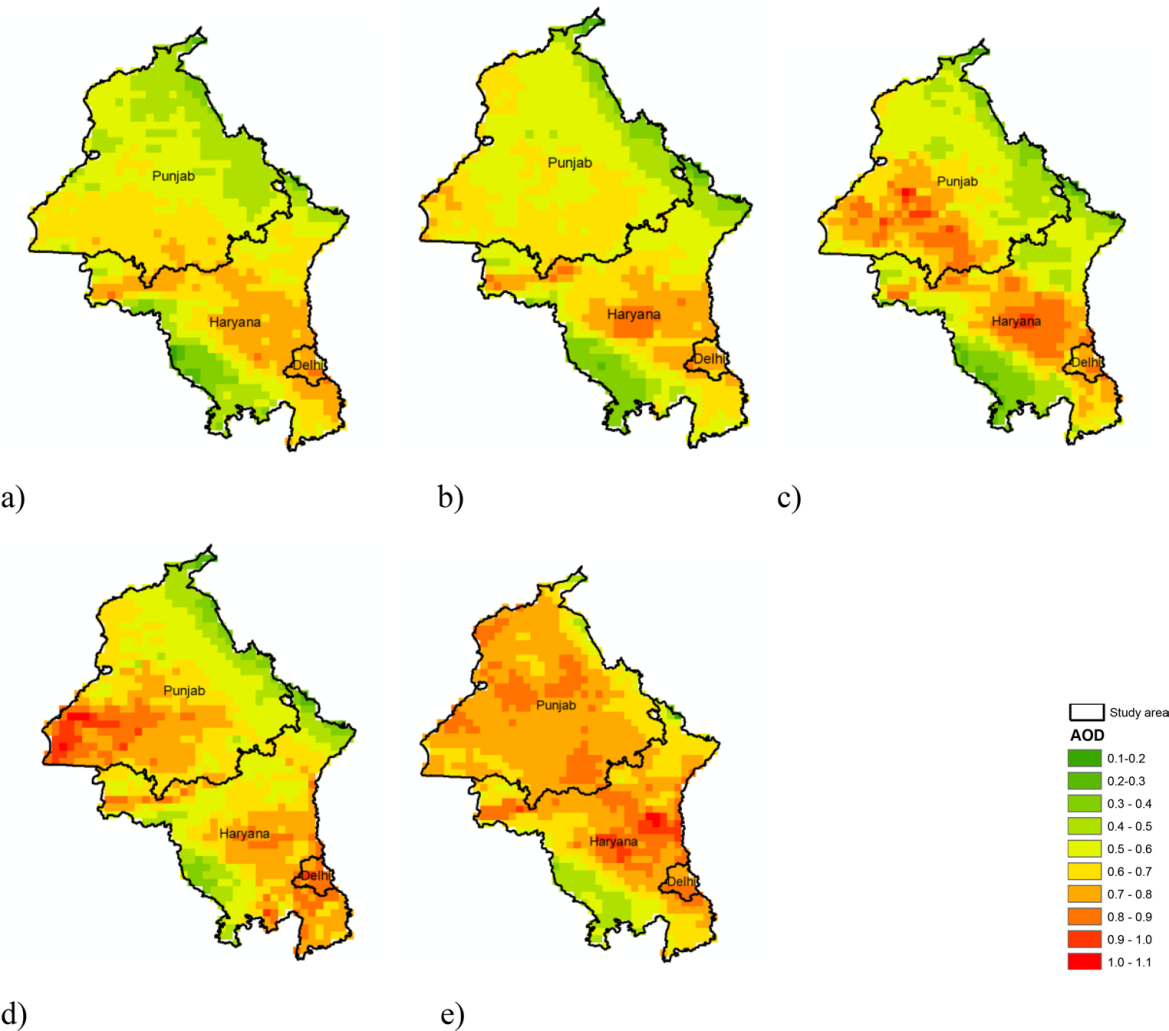
Spatiotemporal variation of post-monsoon Aerosol Optical Depth (AOD) over Delhi and surrounding regions for the years (**a**) 2020, (**b**) 2021, (**c**) 2022, (**d**) 2023, and (**e**) 2024. The maps emphasize seasonal AOD dynamics during the stubble burning period (October–November), revealing interannual differences in aerosol loading and potential transport pathways

The post-monsoon composites (Fig. 4a–e) emphasize the season of maximum haze. Pixels exceeding 0.70 AOD covered 1.42 × 10^5^ ha in 2020, dipped to 1.22 × 10^5^ ha in 2021—coincident with the first round of federal restrictions on stubble burning (Bhuvaneswari et al., 2019)—then more than doubled to 2.62 × 10^5^ ha in 2022 and 3.06 × 10^5^ ha in 2023. The area exploded to 6.02 × 10^5^ ha in 2024, enveloping Delhi and large swaths of south-eastern Punjab and northern Haryana. Escalating fines under the 2024 Amendment Rules (Commission for Air Quality Management, 2024) were evidently insufficient to curb burning intensity.

### Active-fire counts (AFC)

**Fig. 5:**
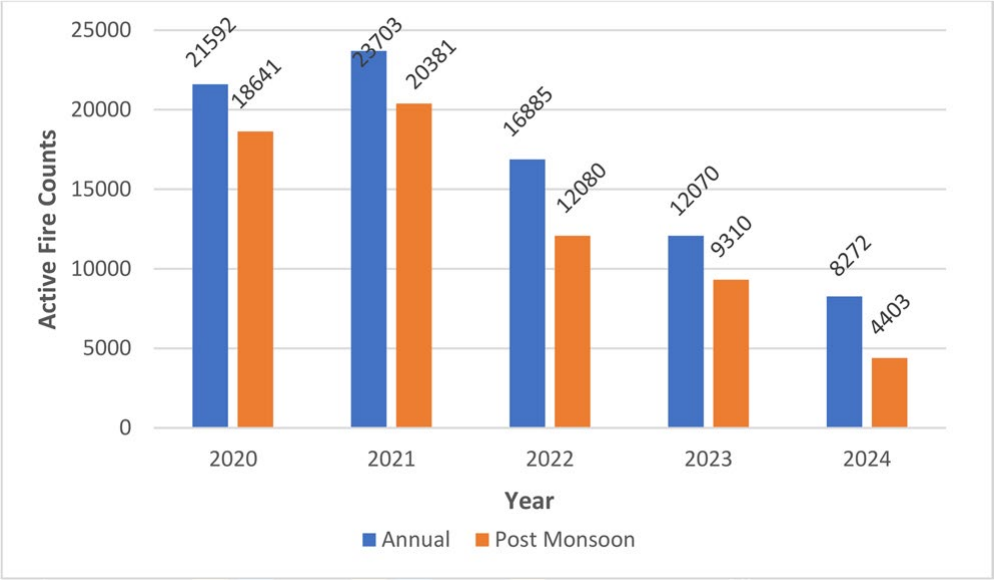
Graphical representation of annual active fire counts detected in the study area from 2020 to 2024. Data derived from VIIRS active fire products highlight temporal fluctuations in fire intensity and frequency, particularly during the post-monsoon season, corresponding to crop residue burning in Punjab and Haryana

**Fig. 6:**
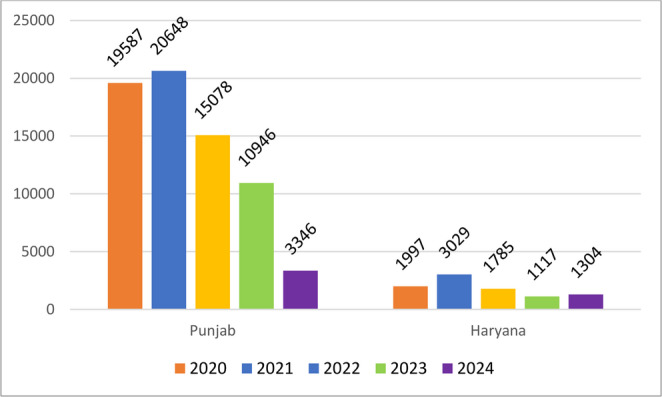
Annual variation in active fire counts across Punjab and Haryana from 2020 to 2024 based on VIIRS satellite observations. The graph illustrates temporal trends in crop residue burning activities, with notable peaks and reductions across years

Total AFC peaked in 2021 (Fig. 5), with ~ 75% of detections occurring in the post-monsoon window. Relative to 2021, study-wide counts fell 28% in 2022, 49% in 2023, and 65% in 2024, mirroring the phased implementation of penalties and subsidy schemes for straw management equipment. State-resolved data (Fig. 6) show Punjab dominating every year; its AFC in 2024 was 83% lower than in 2021, while Haryana’s dropped 56%. Paradoxically, 2024 post-monsoon AOD still rose, consistent with the non-linear relationship between fire counts, PM2.5 and down-wind aerosol load reported by Mangaraj et al. (2025): fewer yet more intense or unfavorably timed burns can still drive high particulate concentrations. As discussed by Mangaraj et al. (2025), the increase in AOD might not only be due to stubble burning but also due to various anthropogenic emissions, dust transport and secondary aerosol formation. Recent modeling efforts in India underscore the complexity of fire–aerosol relationships under changing climate and land-use regimes (Buri et al. 2025).

### Extreme-fire analysis

For 2024, the 98th-percentile brightness-temperature threshold (341 K) isolated 423 extreme fire pixels (5.1% of total detections). Forty-six events fell between 15 and 31 October, coinciding with the steepest NDVI decline. These high-intensity burns serve as temporal anchors for trajectory diagnosis and aerosol build-up assessments. Regional GRACE and remote sensing studies highlight how terrestrial signals, including vegetation and moisture anomalies, co-vary with atmospheric pollution events (Satish Kumar et al. 2021, 2023; Kang and Sridhar 2020). Also, as seen in riverine systems, sediment and aerosol transport are highly sensitive to localized land disturbances, including fire-driven vegetation loss (Nagireddy et al. 2022).

### NDVI dynamics during post-monsoon 2024

**Fig. 7:**
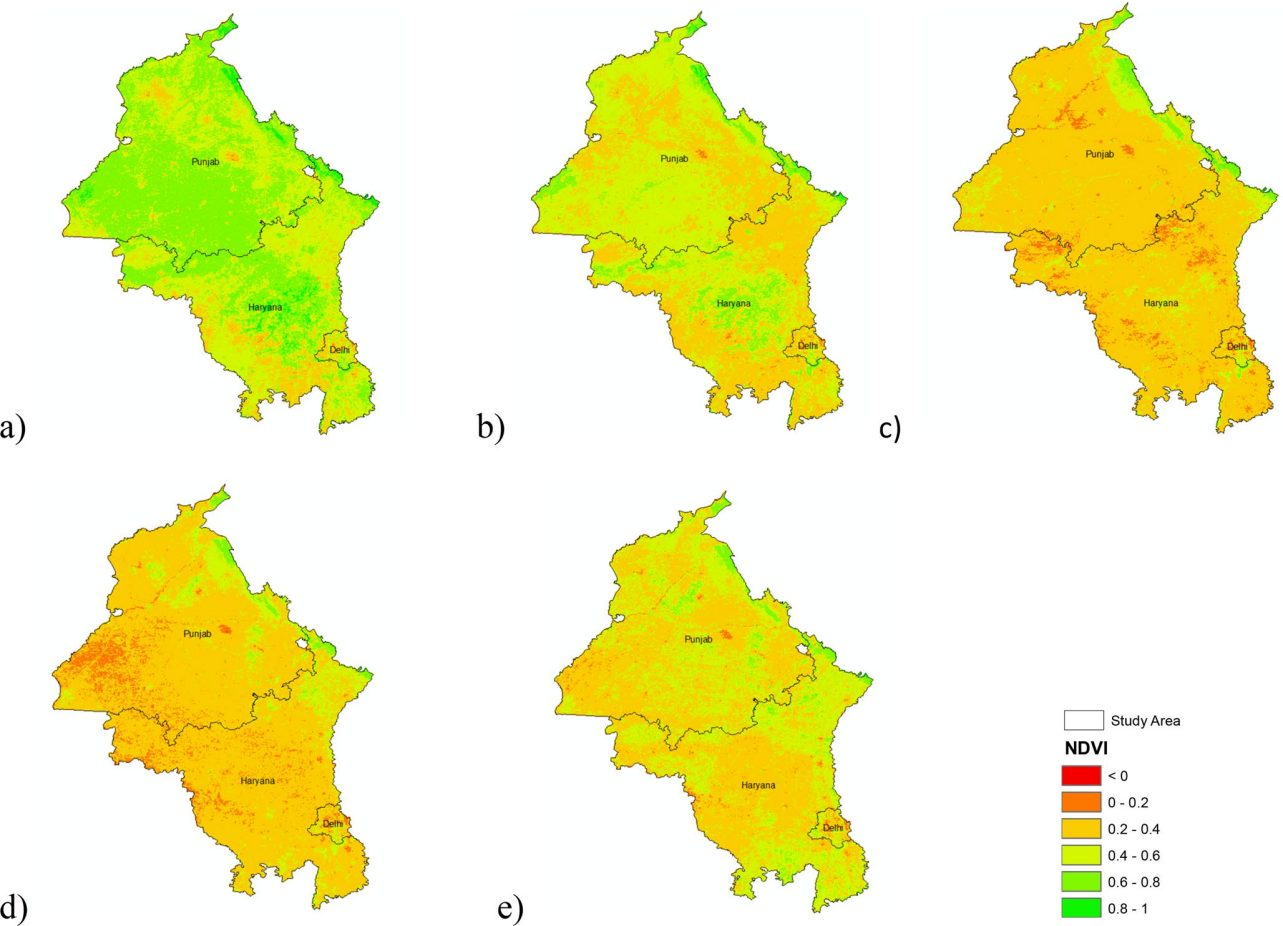
Spatiotemporal variation of post-monsoon Normalized Difference Vegetation Index (NDVI) over the study area on (**a**) September 29, 2024, (**b**) October 15, 2024, (**c**) October 31, 2024, (**d**) November 16, 2024, and (**e**) December 2, 2024. The maps capture the phenological changes in vegetation during the crop residue burning season, reflecting patterns of harvest, field clearing, and regrowth across Punjab, Haryana, and adjoining regions

MODIS NDVI composites (Fig. 7 a–e) show healthy vegetation on 29 September, followed by progressive browning. By 15 October ~ 40% of cropland exhibited NDVI loss; by 31 October the affected area reached 86%, and suppressed greenness persisted through mid-November. Spatiotemporal modeling of vegetation changes across Indian basins supports the linkage between NDVI loss and post-harvest land clearing (Loukika et al. 2023). The rapid decline confirms large-scale paddy harvesting and field clearing. Such phenological signatures, coupled with fire detections, substantiate that open burning—not mechanical removal—is the dominant residue-disposal method (Panjala et al. 2025). Also, these phenological shifts are consistent with modeled vegetation transitions under combined LULC and climate change scenarios (Loukika et al. 2022).

### HYSPLIT trajectory assessment

**Fig. 8:**
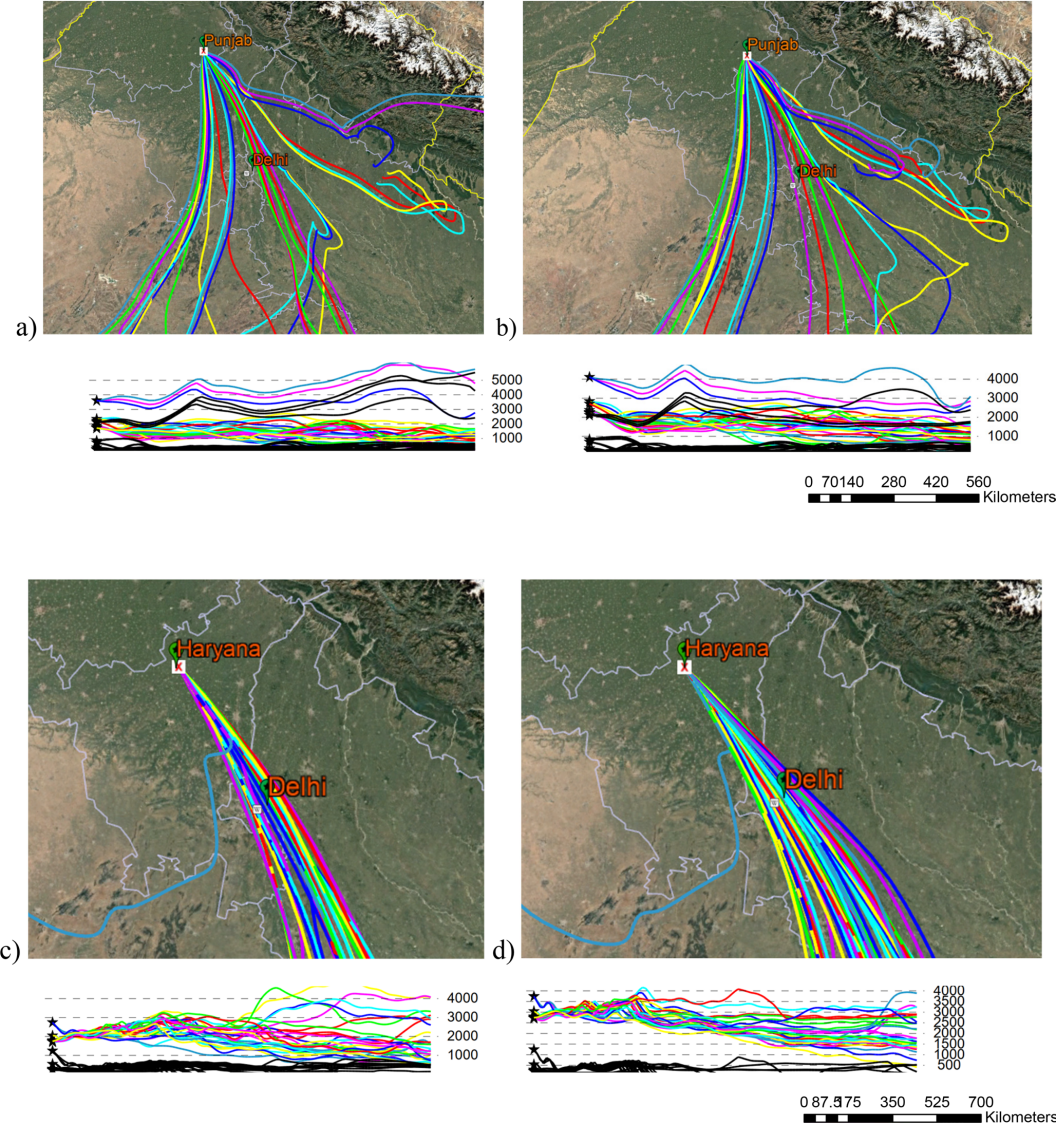
NOAA HYSPLIT forward trajectory simulations originating from (**a**) and (**b**) Punjab and (**c**) and (**d**) Haryana during October 2024. The trajectories illustrate potential atmospheric transport pathways of emissions from stubble burning regions, with plume movements extending toward and surpassing Delhi, indicating likely downwind impact on urban air quality. (Legend shows the elevation in MSL)

**Fig. 9:**
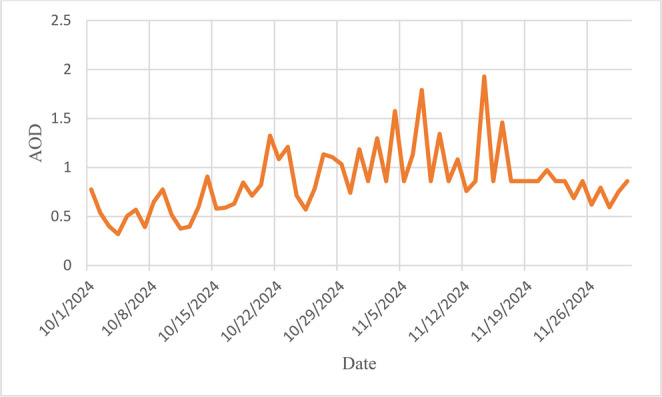
Daily mean Aerosol Optical Depth (AOD) over Delhi during October and November 2024. The time series highlights short-term fluctuations in aerosol loading, with noticeable peaks corresponding to periods of intense stubble burning activity in upwind regions such as Punjab and Haryana

Ensemble forward trajectories for 24–27 October 2024 (Fig. 8) trace air parcels from Punjab and Haryana directly into the Delhi mixing layer within 36–48 h, corroborating long-range smoke transport (Baghel et al. 2023). Concurrently, Delhi’s daily mean AOD (Fig. 9) climbed from ~ 0.60 on 24 October to a peak of ~ 0.95 on 1 November before gradually subsiding. The temporal alignment between trajectory arrivals and AOD escalation, coupled with reduced local fire activity, implicates transported biomass-burning aerosols as the principal driver. Additional inputs—such as Diwali fire-crackers in early November—may have compounded the peak but do not explain the initial rise.

The passage of air parcels from Punjab to Delhi at elevations of 1500 and 2500 m above mean sea level is depicted in Fig. 8 (a and b). For both elevations, the dispersion is about 30% through Delhi. The passage of air parcels from Haryana to Delhi at elevations of 1500 and 2500 m above mean sea level is depicted in Fig. 6(c and d). For both elevations, the dispersion is about 50% through Delhi. Additionally, most of the air parcels from both locations are between 2000 and 3000 m above sea level. In contrast, the air parcels that cause Delhi’s AOD to rise during the final week of October are caused by air from Punjab and Haryana which is in coincidence with the decrease in NDVI during the same period.

Overall, the multi-sensor evidence chain—declining NDVI, clustered extreme fires, coherent back-trajectories, and synchronous AOD surges—confirms that crop-residue burning in Punjab and Haryana remains a dominant episodic source of Delhi’s autumn haze despite recent policy measures. Integrated analyses combining trajectory models and land-surface changes have been effective in tracing environmental shifts in Indian basins (Setti et al. 2020b). Continuous satellite monitoring, stricter enforcement, and accelerated adoption of in-situ straw incorporation are therefore critical to achieving sustained air-quality improvements across the Indo-Gangetic Plain.

## Conclusions

Ground-based monitors and satellite records consistently point to crop-residue burning (CRB) in Punjab and Haryana as a dominant episodic driver of Delhi’s post-monsoon haze. By integrating MODIS 550 nm aerosol optical depth (AOD), VIIRS 375 m active-fire detections, MODIS 16-day NDVI composites, and five-day NOAA-HYSPLIT trajectories for 2020–2024, this study refined that attribution and quantified recent trends. Key outcomes are:

- Persistent high aerosol loading: Pixels with AOD > 0.70 were present every year across the tri-state domain. Although Delhi’s *annual* mean AOD dipped slightly in 2024, its *post-monsoon* mean increased, underscoring the seasonal influence of transported biomass smoke.
- Fire-activity trends: Active-fire counts (AFC) peaked in 2021; by 2024 AFC had fallen 83% in Punjab and 56% in Haryana, reflecting the introduction of enhanced regulatory measures and expanded support for residue-management alternatives. Extreme-fire days—defined by the 98th-percentile brightness-temperature threshold (341 K)—clustered in the third and fourth weeks of October 2024.
- Vegetation phenology as burn proxy: NDVI declined across ~ 86% of cropland between 15 and 31 October 2024, confirming synchronous paddy harvesting and field burning.
- Smoke transport: HYSPLIT forward trajectories for extreme-fire days delivered air parcels from Punjab–Haryana to Delhi within 36–48 h; daily AOD in Delhi rose from ≈ 0.60 to ≈ 0.95 immediately thereafter, producing severe smog episodes in late October and early November 2024.
- The analysis concludes that stubble burning is one of the causes in the transport of pollutants to Delhi which gets amplified with the vehicular emissions and fire-works in the post monsoon season.

The results emphasize the urgency of targeted post-monsoon mitigation. Recommended actions include (i) accelerated adoption of in-situ straw incorporation, baler–mulcher, and bio-energy pathways in hotspot districts; (ii) effective implementation of stubble-burning regulatory frameworks, supported by real-time satellite surveillance and community engagement; and (iii) parallel reductions in urban vehicular and industrial emissions to avoid compounding episodes. Addressing agricultural emissions must consider crop yield resilience and farmer incentives under changing climate scenarios (Debnath et al. 2020). The yearly interpretations between AOD and AFC might be uncertain because of differences in spatial resolution, detection sensitivity, and temporal coverage between the MODIS-based AOD and active fire datasets. In addition to this, the current study does not concentrate on the contribution of the individual aerosol sources that limits direct attribution to observed AOD in the region.

Future work should couple CRB inventories with high-resolution meteorological drivers (e.g., WRF-GFS) and chemical transport models to resolve synergistic effects among regional fires, boundary-layer dynamics, and local sources that shape Delhi’s extreme air-quality events. Fire radiative power (FRP) could be included in future studies to give a more reliable indicator of emissions. A thorough climatological comparison was outside the current research, but similar meteorological anomalies, like wind stagnation during the 2024 post-monsoon period helps in expanding such analyses to long-term circulation and ventilation patterns would further improve attribution of aerosol loading.

## Data Availability

Datasets will be made available upon request to the authors.
